# Iterative Genome Engineering Platform Enables Efficient Sucrose Biosynthesis From CO_2_ in Photosynthetic *Synechococcus elongatus* UTEX 2973

**DOI:** 10.1111/pbi.70702

**Published:** 2026-06-15

**Authors:** Shubin Li, Tao Sun, Dailin Liu, Tong Zhang, Lei Chen, Weiwen Zhang

**Affiliations:** ^1^ School of Synthetic Biology and Biomanufacturing Tianjin University Tianjin People's Republic of China; ^2^ Frontier Science Center for Synthetic Biology and Key Laboratory of Systems Bioengineering Ministry of Education of China Tianjin People's Republic of China; ^3^ State Key Laboratory of Synthetic Biology Tianjin University Tianjin People's Republic of China; ^4^ Center for Biosafety Research and Strategy Tianjin University Tianjin People's Republic of China; ^5^ Haihe Laboratory of Sustainable Chemical Transformations Tianjin China

**Keywords:** genome instability, homologous recombination, iterative genetic engineering, single crossover, sucrose production

## Abstract

The single crossover occurring via homologous recombination is a common phenomenon existing among microbes like *Escherichia coli* , *Bacillus subtilis* , *Vibrio natriegens* , *Gluconobacter oxydans* and most cyanobacteria species, threatening the stability of engineered strains and challenging iterative genetic engineering. Among them, we take the fast‐growing cyanobacterium *Synechococcus elongatus* UTEX 2973 (Syn2973) as a representative study due to its promising roles for CO_2_ fixation and bioconversion. We established three marker‐free platforms to achieve stable genome recombination: (i) T4CROSS, which employs two plasmids and four rounds of single crossover; (ii) TRIPLEARM, which uses a single plasmid containing three homologous arms for three rounds of single crossover; and (iii) CRISPRARM, which integrates CRISPR/Cpf1‐mediated genome editing with homologous recombination. As proof of concept, we employed the CRISPRARM platform for a three‐step sequential engineering of the sucrose biosynthetic pathway. The final engineered strain produced 7.12 g L^−1^ of sucrose within 4 days.

## 1 Introduction

Homologous recombination is a widely used strategy for precise genome modification in microbes, enabling accurate and site‐specific exchange of genetic material between homologous DNA sequences (Yu et al. 2000). Typically, a suicide vector carrying the desired cargo cassette flanked by homologous arms is introduced into the target microbe via electroporation or conjugation. The recombination process ideally proceeds through two sequential crossover events: an initial single crossover, followed by a second recombination event that results in a double crossover and stable genomic integration. However, in many cases including *Lactococcus lactis* (Lu et al. 2017), *Clostridium acetobutylicum* (Heap et al. 2012), *Anabaena* (*Nostoc*) (Gibbons et al. 2022) and etc., the second crossover is inefficient, leading to the persistence of single‐crossover intermediates. This partial integration is problematic, as it compromises the genetic stability of engineered strains and hinders applications such as clean gene knockouts or iterative genome modifications. Moreover, in polyploid microbes like cyanobacteria (Griese et al. 2011), haloarchaea (Ludt and Soppa 2019) and *Azotobacter vinelandii* (Maldonado et al. 1994), residual plasmid backbone sequences retained after single crossover events may serve as unintended homologous targets in subsequent integrations, further complicating genetic engineering efforts.

In this study, we first demonstrated that single‐crossover events during homologous recombination occur in multiple bacteria including *Escherichia coli* , *Bacillus subtilis* , *Vibrio natriegens* , *Gluconobacter oxydans* and the fast‐growing cyanobacterium *Synechococcus elongatus* UTEX 2973 (Syn2973). We then focused on the study of Syn2973 due to two main reasons: (i) cyanobacteria are the only prokaryotes capable of oxygenic photosynthesis, contributing 20%–30% of global CO_2_ fixation (Rosgaard et al. 2012). This unique capability makes them attractive chassis for developing photosynthetic cell factories, especially in the pursuit of carbon neutrality. Syn2973 exhibits a significantly shorter doubling time of just 1.5 h under optimal conditions (Ungerer et al. 2018) along with enhanced tolerance to high light intensities (up to 1500 μmol photons m^−2^ s^−1^) and elevated temperatures (up to 42°C). Additionally, Syn2973 can reach a dry cell weight of 23.41 g L^−1^ under semi‐continuous cultivation, with a productivity of 2.4 g L^−1^ day^−1^ (Long et al. 2022). Its potential as a photosynthetic production platform has been demonstrated, with engineered strains achieving efficient production of sucrose (Lin et al. 2020), inositol (Sun et al. 2023) and amino acids (Dookeran and Nielsen 2021), etc. and (ii) Syn2973 is both polyploid and exhibits limited efficiency in achieving double crossovers following single‐crossover recombination. These characteristics not only make iterative genetic engineering particularly challenging in this strain (Wang et al. 2025; Chen et al. 2021)，but also mean that resolving these issues could provide broadly applicable strategies for addressing similar limitations in other microbes.

**FIGURE 1:**
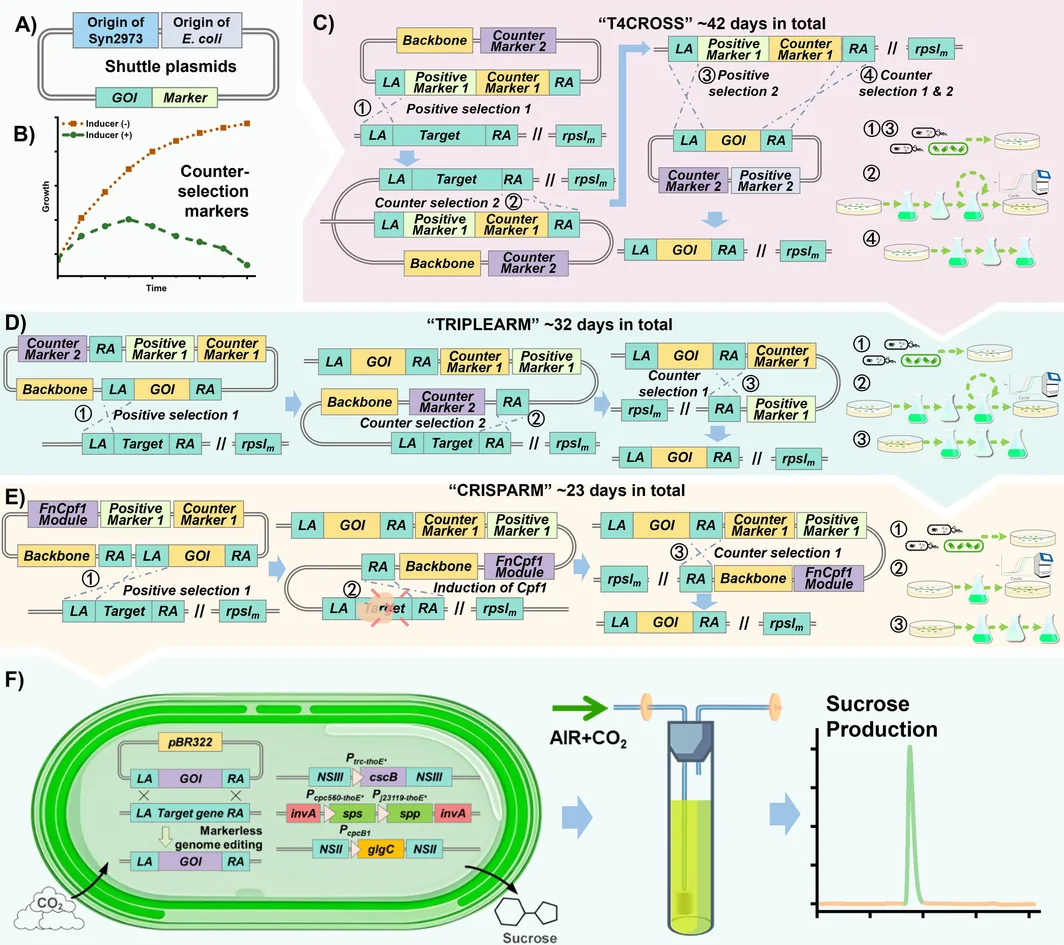
Schematic overview of this study. (A) Diagram of the shuttle vector structure. (B) Illustration of the growth effects of the counter‐selectable marker before and after induction. (C) Schematic representation of the recombination strategy and experimental procedure of the ‘T4CROSS’ method. Circled numbers indicate the sequence of the four single crossover events. (D) Recombination strategy and experimental procedure of the ‘TRIPLEARM’ method. Circled numbers indicate the sequence of the three single crossover events. (E) Recombination strategy and experimental procedure of the ‘CRISPARM’ method. Circled numbers indicate the sequence of the three single crossover events. (F) Iterative scarless genome editing using the developed methods to construct a sucrose‐producing *Synechococcus elongatus* UTEX 2973 strain, and a schematic diagram of sucrose production in a photobioreactor.

To address the limitations, we systematically optimized marker‐free genetic manipulation strategies in Syn2973 from the following aspects: (i) Optimization of stable shuttle plasmids (Figure 1A): We constructed two shuttle vectors, pSES and pSEL, based on the endogenous plasmids pANS and pANL of Syn2973. In addition, a re‐engineered plasmid (pRSF), derived from the RSF1010 origin of replication, was developed to enhance transformation efficiency. (ii) Screening of effective counter‐selection markers (Figure 1B): We evaluated four commonly used counter‐selection markers—*sacB* (Pelicic et al. 1996), *rpsl*_*12*_ (Takahama et al. 2004), *sepT*_*2*_ (Zhou et al. 2019) and *tetA* (Stavropoulos and Strathdee 2000)—in Syn2973. Among them, *rpsl*_*12*_ and *sepT*_*2*_ demonstrated reliable performance in enabling counter‐selection. (iii) Development of marker‐free genetic manipulation tools (Figure 1C–E): Three novel strategies were established for efficient, marker‐free genome editing in fast‐growing cyanobacterium Syn2973: T4CROSS, which employs two plasmids for four rounds of single‐crossover recombination; TRIPLEARM, which uses a single plasmid containing three homologous arms to facilitate iterative recombination; CRISPRARM, which combines CRISPR/Cpf1 with homologous recombination to enable precise genome modifications. (iv) Iterative marker‐free gene editing in Syn2973 (Figure 1F): Using sucrose biosynthesis as a proof‐of‐concept, we applied the CRISPRARM system in a three‐step iterative editing workflow. The resulting strain achieved a sucrose titre of 7.12 g L^−1^ within 4 days, demonstrating both the feasibility and high efficiency of the system for sequential genome engineering in fast‐growing cyanobacterium Syn973.

## 2 Methods

### 2.1 Bacterial Strains and Growth Conditions

The wild‐type Syn2973 (hereafter referred to as WT) and engineered strains were cultivated in standard BG11 medium (pH 7.5) (Stanier et al. 1971). For liquid cultures, cells were grown in shaking flasks using an illuminated incubator (HNYC‐202T, Honour, Tianjin, China) at 37°C with agitation at 200 rpm and continuous illumination of ~500 μmol photons m^−2^ s^−1^. For solid cultures, plates were incubated at 37°C under a light intensity of ~300 μmol photons m^−2^ s^−1^ in a growth chamber (SPX‐250B‐G, Boxun, Shanghai, China). *E. coli* strains were cultured under similar temperature and shaking conditions but without light exposure. *B. subtilis* strains were cultured under similar conditions of *E. coli*. SMM medium (2.0 g/L (NH_4_)_2_SO_4_, 14.0 g/L K_2_HPO_4_, 6.0 g/L KH_2_PO_4_, 0.2 g/L MgSO_4_, 1.0 g/L sodium citrate dihydrate), MM competence medium (10 mL/L SMM medium, 125 μL/L 40% glucose, 100 μL/L tryptophane solution at a concentration of 2 mg/mL, 60 μL/L 1 M MgSO_4_, 10 μL/L 20% Casamino acids, 5 μL/L 0.22% ammonium ferric citrate), and starvation medium (10 mL/L SMM medium, 125 μL/L 40% glucose, 60 μL/L 1 M MgSO_4_) were used during transformation. The growth conditions of *Vibrio natriegens* strains are largely comparable to those of *E. coli* , with the exception that LB2 medium (standard LB medium with additional 1.5% NaCl) is used for cultivation. *G. oxydans* strains were cultivated at 30°C and 200 rpm in mannitol medium (5 g/L yeast extract, 3 g/L peptone and 50 mM mannitol). When appropriate, antibiotics were added to the media at the following final concentrations: for Syn2973, 20 μg mL^−1^ kanamycin, 20 μg mL^−1^ chloramphenicol, 50 μg mL^−1^ spectinomycin, 50 μg mL^−1^ erythromycin and/or 50 μg mL^−1^ streptomycin; for *E. coli* , 100 μg mL^−1^ ampicillin, 50 μg mL^−1^ kanamycin, 50 μg mL^−1^ chloramphenicol, 100 μg mL^−1^ spectinomycin, 200 μg mL^−1^ erythromycin, and/or 50 μg mL^−1^ streptomycin; for *Bacillus subtilis* , 5 μg mL^−1^ chloramphenicol; for *Vibrio natriegens* , 100 μg mL^−1^ ampicillin, 200 μg mL^−1^ kanamycin, 5 μg mL^−1^ chloramphenicol; for *G. oxydans* , 50 μg mL^−1^ kanamycin, 50 μg mL^−1^ cefoxitin. When required, 50 μg mL^−1^streptomycin, 1 mM IPTG (isopropyl β‐D‐1‐thiogalactopyranoside), or 2 mM theophylline was used as an inducer. Optical density was measured at 750 nm (OD_750_) for Syn2973 and at 600 nm (OD_600_) for other bacterium using a UV‐1750 spectrophotometer (Shimadzu, Kyoto, Japan).

### 2.2 Plasmids and Strains Construction

*Escherichia coli* DH5α was used for plasmid construction and amplification. All the primers and DNA fragments were chemically synthesized by GENEWIZ Inc. (Suzhou, China). In this study, plasmid construction was mainly carried out using ClonExpress MultiS One Step Cloning Kit (Vazyme Biotech Co. Ltd., Nanjing, China) and Golden Gate assembly technology. The constructed plasmids, their construction methods, and the corresponding primers used were listed in Table S1, among which the synthetic DNA sequences used for plasmid construction are listed in Table S2. The primers used for PCR or qPCR verification are listed in Table S3 and some of the key plasmid sequences are listed in Table S4. The transformation of Syn2973 and Syn7942 was carried out through conjugation according to studies reported previously (Yunus et al. 2018). When testing transformation efficiency, the initial Syn2973 input was controlled at an appropriate level, where volume × OD_750nm_ = 0.01, 0.1 or 0.5 (approximately equals to 1.45 × 10^6^, 1.45 × 10^7^ or 7.25 × 10^7^ cells). The genome editing strategy in Syn7942 was similar to that used in strain 2973, except that the light intensity was changed to 50 μmol photons m^−2^ s^−1^.

For genomic insertion in *E. coli* , the λ Red recombination method (Yu et al. 2000) was employed. *E. coli* BW25113 harbouring the plasmid pKD46 was activated at 30°C. The overnight culture was diluted 1:100 into fresh medium and induced with 1% (w/v) l‐arabinose. When the culture reached an OD_600_ of 0.5–0.6, the cells were washed twice with ice‐cold ultrapure water and finally resuspended in 1/20 of the induction volume with ultrapure water. A total of 100 μL of the competent cells was mixed with 5 μL of plasmid DNA (50 ng/μL) and transferred into a pre‐chilled 1 mm electroporation cuvette. Electroporation was performed at 1800 V, after which 1 mL of LB medium was immediately added for recovery. Then the cells were incubated at 30°C for 2 h with shaking, followed by centrifugation and plating onto selective agar plates. The plates were then incubated at 30°C for 24 h.

For marker‐less genome editing in *E. coli* , the T4CROSS method was applied. First, the pR6K‐eco‐lacZ‐km plasmid was introduced via λRed recombination, followed by plating and verification of single colonies. Verified colonies were cultured overnight at 30°C, then inoculated into fresh medium at a 1:100 ratio. Induction was carried out with 0.5 mM IPTG and 2 mM theophylline to promote double‐crossover integration. After 12 h of growth, when the culture became fully turbid, cells were streaked to isolate single colonies for verification and subsequent inoculation. Next, the pR6K‐eco‐lacZ‐in plasmid was introduced using the same λRed recombination procedure. Verified colonies were cultured overnight and reinoculated (1:100) into fresh medium. After 30 min of growth, 0.5 mM IPTG, 2 mM theophylline, and 5% sucrose were added simultaneously to induce the formation of the final scarless mutants. After 12–18 h, when the culture became turbid, cells were streaked for single colonies and verified. For the TRIPLEARM method, the pR6K3‐eco‐lacZ‐in plasmid was introduced via λRed recombination. Double‐crossover integration was first induced using 0.5 mM IPTG and 2 mM theophylline, followed by induction with 5% sucrose to generate scarless mutants. The detailed procedure was similar to that described above. For the CRISPRARM method, the pCPF3‐eco‐lacZ‐sacB plasmid was introduced using λRed recombination. Expression of Cpf1 was induced with 0.5 mM IPTG and 2 mM theophylline to facilitate double‐crossover recombination, followed by induction with 5% sucrose to obtain scarless mutants. The procedure was similar to the methods described above.

For *B. subtilis* , strains were first activated from glycerol stocks on LB agar plates, and a single colony was inoculated into MM competence medium for overnight culture. Then, 0.6 mL of the overnight culture was added to 10 mL of fresh MM competence medium and incubated for 3 h at 37°C, followed by supplementation with 10 mL prewarmed starvation medium for an additional 2 h. 0.4 mL culture were mixed with DNA and incubated for 1 h at 37°C with shaking. Cells were pelleted, resuspended and plated on selective agar plates.

Transformation of *G. oxydans* was performed via conjugation. Donor strains carrying the plasmid of interest, helper strains harbouring pRL443 and recipient strains were separately cultured to mid‐log phase. Each culture (10 mL) was washed three times with mannitol medium, resuspended in 100 μL of the same medium, and then mixed together. The mixture was spread onto mannitol solid medium lacking antibiotics and incubated at 30°C for 24 h. Cells were subsequently washed off the plates with mannitol medium and plated at 1:10, 1:100 and 1:1000 dilutions onto plates containing the appropriate antibiotics.

For marker‐less genome editing in *G. oxydans* , the T4CROSS method was employed. First, the pR6K‐gox‐tyb‐km plasmid was introduced, followed by plating and verification of single colonies. Verified colonies were inoculated into 10 mL fresh mannitol medium and cultured at 30°C for 2–3 days until turbidity was observed. The culture was then inoculated into fresh medium at a ratio of 1:100 and grown for 1 day, after which 2 mM theophylline was added to induce double‐crossover integration. After 2–3 days of growth, when the culture became fully turbid, cells were streaked to isolate single colonies for verification and subsequent inoculation. Next, the pR6K‐gox‐tyb‐in plasmid was introduced. Verified colonies were inoculated into 10 mL fresh mannitol medium and cultured at 30°C for 2–3 days until turbid. The culture was then transferred (1:100) into fresh medium and grown for 1 day, followed by simultaneous induction with 2 mM theophylline and 5% sucrose to generate the final scarless mutants. After 2–3 days, when the culture became turbid, cells were streaked for single colonies and verified. For the TRIPLEARM method, the pR6K3‐gox‐tyb‐in plasmid was introduced. Double‐crossover integration was first induced using 2 mM theophylline, followed by induction with 5% sucrose to generate scarless mutants. The detailed procedure was similar to that described above. For the CRISPRARM method, the pCPF3‐gox‐tyb‐sacB plasmid was introduced. Expression of Cpf1 was induced with 0.5 mM IPTG and 2 mM theophylline to facilitate double‐crossover recombination, followed by induction with 5% sucrose to obtain scarless mutants. The procedure was similar to the methods described above.

Transformation of *V. natriegens* was performed via conjugation. To enable selection of *E. coli* and *V. natriegens* , a pMMB plasmid conferring ampicillin and kanamycin resistance was first introduced into *V. natriegens* by electroporation (Electroporation was performed using 1 M sorbitol as the protective buffer, with conditions of 0.4 kV, 1 kΩ and 25 μF.) to generate the recipient strain. Donor strains carrying the plasmid of interest, helper strains harbouring pRL443, and the recipient strain were separately cultured to mid‐log phase. Each 10 mL culture was washed three times with LB medium, resuspended in 100 μL of LB, and mixed together. The mixture was spread onto LB agar plates without antibiotics and incubated at 37°C for 24 h. Cells were then washed off the plates with LB medium and plated at 1:10, 1:100 and 1:1000 dilutions onto plates containing the appropriate antibiotics. The strains used in this study were listed in Table S5.

### 2.3 Natural Transformation of Syn2973

For natural transformation (Barten and Lill 1995), 1 mL of exponentially growing Syn2973 culture (OD_750_ = 1.0) was collected by centrifugation and resuspended in 50 μL of BG11 medium containing 500 ng of linear DNA. The mixture was then incubated statically at 37°C under a light intensity of 50 μmol photons m^−2^ s^−1^ for 5 h, with gentle shaking every 2.5 h. Finally, the mixture was evenly spread on sterile filters (0.45 μm pore size) coated on BG11 agar plates containing the appropriate antibiotics and cultured under a light intensity of approximately 200 μmol photons m^−2^ s^−1^.

### 2.4 Plasmid Stability Test

Plasmid stability was tested according to previously described methods (Márquez and García 2017). The strain was centrifuged at 4000 rpm and 4°C then resuspended in BG11 medium. This process was repeated twice. Subsequently, continuous cultivation was carried out in BG11 medium without antibiotics starting from an initial OD_750_ of 0.1. Each time the OD_750_ of the culture reached 1, the above process was repeated. For each round, strains with an OD_750nm_ of 1.0 (approximately equals to 1.45 × 10^8^ cells) were diluted by 1000‐fold and spread onto antibiotic‐free BG11 plates. Fifty single colonies obtained were then subjected to resistance testing.

### 2.5 *β*‐Galactosidase Activity Measurement

The method for measuring β‐galactosidase activity is based on a previous study (Li et al. 2018). In simple terms, cells from the exponential growth phase of Syn2973 (volume × OD_750nm_ = 1, approximately equals to 1.45 × 10^8^ cells) are centrifuged and resuspended in 1 mL of Z buffer (containing 60 mM Na_2_HPO_4_, 40 mM NaH_2_PO_4_, 10 mM KCl, 1 mM MgSO_4_ and 40 mM β‐mercaptoethanol). Then, 50 μL of 0.1% SDS and 50 μL of chloroform are added to lyse the cells. Subsequently, 200 μL of ortho‐nitrophenyl‐beta‐d‐galactopyranoside (ONPG; 4 g L^−1^) is added to initiate the reaction. The reaction mixture is incubated for a certain period at 30°C and 750 rpm in a constant temperature shaker for 3 min. Finally, 500 μL of 1 M Na_2_CO_3_ is added to stop the reaction. The Miller value is calculated using the OD_420_ of the supernatant (Miller = 1000 × OD_420_/3).

### 2.6 *β*‐Glucuronidase Activity Measurement

The method for measuring β‐glucuronidase activity is based on a previous study (Zhou et al. 2019). In simple terms, cells from the exponential growth phase of Syn2973 (volume × OD_750nm_ = 1, approximately equals 1.45 × 10^8^ cells) are centrifuged and resuspended in 1 mL of Z buffer (containing 60 mM Na_2_HPO_4_, 40 mM NaH_2_PO_4_, 10 mM KCl, 1 mM MgSO_4_ and 40 mM β‐mercaptoethanol). Then, 50 μL of 0.1% SDS and 50 μL of chloroform are added to lyse the cells. Subsequently, 200 μL of p‐nitrophenyl‐β‐D‐glucuronide (PNPG; 4 g L^−1^) is added to initiate the reaction. The reaction mixture is incubated for a certain period at 30°C and 750 rpm in a constant temperature shaker for 3 min. Finally, 500 μL of 1 M Na_2_CO_3_ is added to stop the reaction. The activity value is calculated using the OD_420_ of the supernatant (1000 × OD_420_/3).

### 2.7 QRT‐PCR Analysis

The determination of copy numbers and the assessment of genome homozygosity are performed using qRT‐PCR (Cantsilieris et al. 2013). After growing for 48 h, the culture (volume × OD_750_ = 2, approximately equals to 2.9 × 10^8^ cells) was collected and the DNA was extracted through a TIANamp Bacteria DNA Kit (TIANGE, Beijing, China). QRT‐PCR reactions were performed using PowerUp SYBR Green Master Mix (Thermo Fisher Scientific, MA, USA) in a 10 μL reaction volume, consisting of 5 μL of the mixture, 3 μL of ddH_2_O, 1 μL of template (diluted to 10 ng μL^−1^ of gDNA) and 0.5 μL of each primer. The reactions were carried out using the StepOnePlus Real‐Time PCR System (Applied Biosystems, CA, USA). Data analysis was conducted using StepOnePlus Analysis Software (Applied Biosystems, CA, USA). The relative gene copy numbers were calculated using the $2^{-∆∆C_{t}}$ method (Livak and Schmittgen 2001), with the 16S rDNA gene (Two copies per genome) serving as an internal reference. Three biological replicates were performed for each condition.

### 2.8 Sucrose Measurement

The concentrate of sucrose was quantified from culture supernatants using high‐performance liquid chromatography (HPLC) on an Agilent 1260 instrument equipped with an Aminex HPX‐87C column (300 mm × 7.8 mm; Bio‐Rad, CA, USA) (Picha 1985). Ultra‐pure water was used as the mobile phase at 80°C and a flow rate of 0.6 mL min^−1^ in isocratic mode. Compounds were detected and quantified from 10 μL sample injections using a refractive index detector. Reported metabolite concentrations represent the average of three biological replicates. Standard products were purchased from Aladdin Scientific. The sucrose standard curve is shown in Figure S1.

### 2.9 Cultivation of Syn2973 Using Bubble Column Bioreactor

The high‐density cultivation of Syn2973 is conducted in 100 mL flat‐bottom glass tubes with a diameter of 30 mm, in a volume of 60 mL of 5 × BG optimized medium (Włodarczyk et al. 2020). Air and carbon dioxide flow rates and ratios (1%, 3% and 5% CO_2_ in air) were controlled using gas mass flow controllers. The total gas flow rate in the tube was maintained at a ratio of 1:1 to the culture volume per minute. Light intensity (500 or 2000 μmol photons m^−2^ s^−1^) and temperature were controlled externally using LEDs and circulating water, respectively.

### 2.10 Statistical Analysis

In this study, ‘all colonies’ refers to 20 independent transformants collected after transformation; with three biological replicates, a total of 60 transformants were analysed in subsequent experiments. ‘Survived colonies’ is defined as the proportion of transformants that remained viable after serial passaging and counter‐selection induction (e.g., Negative selection marker or Cpf1 induction). For downstream analysis, cultures of correctly identified survived colonies were pooled, plated to isolate single colonies, and 20 colonies were randomly selected to calculate the proportions of ‘positive’ and ‘homozygous’ clones among the survived colonies in each replicate. The values shown in the figures represent the proportions of ‘positive’ and ‘homozygous’ clones relative to all colonies, after normalization and error analysis across replicates. Finally, the terms ‘positive’ and ‘homozygous’ are used interchangeably to denote the desired genotype. For haploid hosts ( *E. coli* , *G. oxydans* ), this refers to verified transformants carrying the intended genomic edit. For polyploid hosts (Syn7942, Syn2973), it specifically refers to fully segregated homozygous clones in which all chromosomal copies have been edited, as confirmed by PCR or sequencing, with no detectable wild‐type alleles.

## 3 Results

### 3.1 Characterization of Single‐Crossover Events Across Bacteria and Establishment of Shuttle Plasmids in Syn2973

**FIGURE 2:**
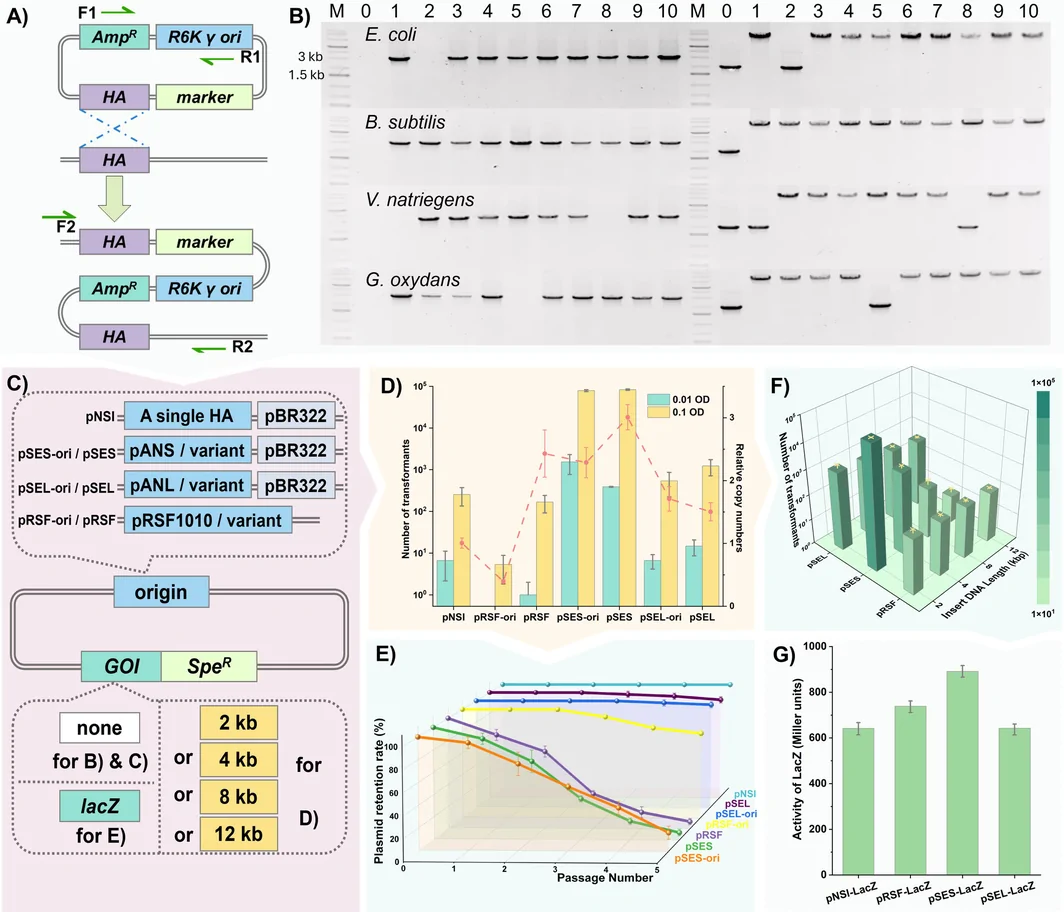
Construction and characterization of shuttle plasmids. (A) Schematic overview of single homologous recombination plasmid design for testing. (B) PCR verification of genomic single‐crossover homologous recombination. The left panel shows the PCR verification results using primers F1 and R1 from panel A, while the right panel shows the results using primers F2 and R2. Lane 0 serves as the control. (C) Schematic overview of shuttle plasmid design and testing. The dashed box at the top shows the replication/integration elements being tested, while the lower dashed box indicates the insertion of the gene of interest (GOI) in panels C–F. (D) Transformation efficiency of different synthetic shuttle plasmids at initial OD of 0.01 and 0.1, along with their relative copy numbers compared to the genome in Syn2973. (E) Relative plasmid retention rates of various synthetic shuttle plasmids during serial passaging without antibiotic pressure. (F) Transformation efficiencies of pSES, pSEL and pRSF plasmids carrying gene fragments of varying lengths (2 kb, 4 kb, 8 kb and 12 kb), with an initial OD of 0.1. (G) Activity levels of β‐galactosidase encoded by pSES, pSEL and pRSF, and comparison to activity from integration at the NSI locus. Error bars represent standard deviations from three independent replicates.

To validate the prevalence of single crossover events, we first examined multiple representative bacteria, including *E. coli* BW25113, *B. subtilis* 168, *V. natriegens* Vmax, *G. oxydans* 621 h and Syn2973. For each species, we introduced a non‐replicative plasmid carrying a single homologous arm and an antibiotic selection marker (Figure 2A). Transformants were successfully obtained in all cases and verified by PCR (Figure 2B), confirming that single crossover recombination is a widespread phenomenon among bacteria. We focused on Syn2973 to address its genetic limitations in this study. Syn2973 is not naturally competent due to the incomplete *pilMNOQ* gene cluster involved in pilus biogenesis (Li et al. 2018), and its transformation typically relies on *E. coli* ‐mediated conjugation. Therefore, shuttle plasmids capable of replication in both *E. coli* and Syn2973 are essential for genetic manipulation. In this study, we focused on two endogenous plasmids from Syn2973—designated pANS and pANL (Chen et al. 2016; Encinas et al. 2014). We constructed shuttle plasmids by combining these origins with the *E. coli* ‐compatible pBR322 backbone, resulting in four variants: pSES‐ori and pSEL‐ori (containing the full reported replication regions), and pSES and pSEL (with *Bsm*BI/*Bsa*I restriction sites removed to support Golden Gate Assembly, along with non‐essential sequence deletions) (Figure 2C, Table S4). As controls, we included pRSF‐ori (based on the RSF1010 broad‐host‐range replicon (Taton et al. 2014)) and pNSI (a pBR322‐derived plasmid containing a single homologous arm to support single crossover integration in Syn2973). As shown in Figure 2D and Figure S2, pSES‐ori showed the highest efficiency. Meanwhile, the modified versions (pSES and pSEL) displayed comparable transformation efficiencies. In contrast, pRSF‐ori showed poor compatibility, which could be due to its lower copy numbers (Figure 2D). Deletion of the suppressor protein RepF of pRSF‐ori (Meyer 2009) (the plasmid pRSF) slightly improved transformation efficiency (Figure 2D). We next assessed plasmid stability, insert capacity, and gene expression performance. After five passages without antibiotic selection, retention rates of pSEL, pSEL‐ori and pRSF‐ori were 93.3%, 96.7% and 78.3%, respectively, indicating good plasmid stability (Figure 2E). To assess fragment tolerance, we inserted 2 kb, 4 kb, 8 kb and 12 kb sequences into pSES, pSEL and pRSF. All three vectors successfully carried up to 12 kb inserts (Figure 2F). Using a *lacZ* reporter gene under the control of the Ptrc promoter, we found that plasmids with higher copy numbers (pSES and pRSF) exhibited stronger β‐galactosidase activity than pSEL (Figure 2E). Taken together, pSES and pSEL were selected as the primary shuttle plasmids for subsequent genetic manipulations in Syn2973 due to their high efficiency, stability and performance.

### 3.2 Development of Efficient Counter‐Selection Systems and Marker‐Free Genetic Tools

**FIGURE 3:**
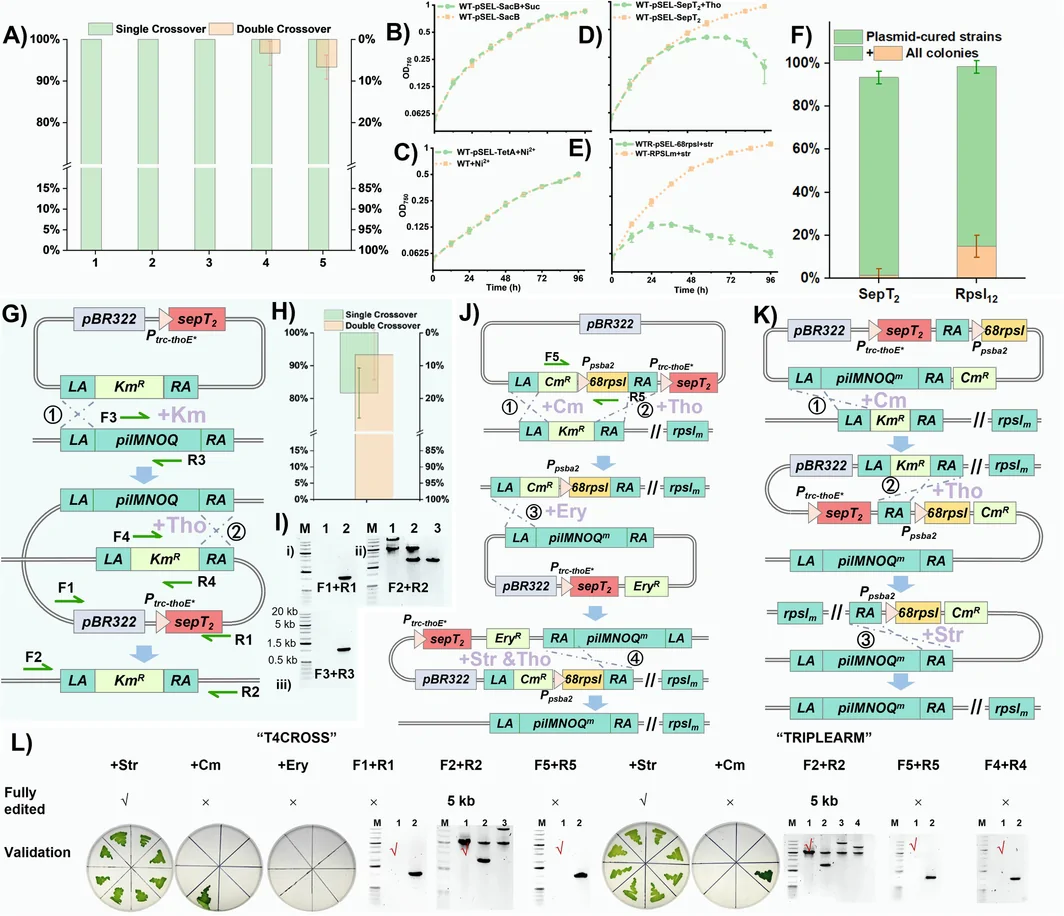
Identification and application of negative selection markers. (A) Proportion of Syn2973 transformants undergoing single‐crossover and double‐crossover recombination across five consecutive passages. (B–E) Effects of different negative selection systems on Syn2973 growth, with or without inducers: (B) SacB system (50 g L^−1^ sucrose), (C) TetA system (2.5 μM Ni^2+^), (D) SepT_2_ system (2 mM theophylline) and (E) RpsL_12_ system (50 μg mL^−1^ streptomycin). (F) Application of SepT_2_ and RpsL_12_ for plasmid curing. The bar graph shows the proportions of plasmid‐free versus plasmid‐retaining colonies (20 colonies per group, 3 groups total) after inducer treatment. Error bars represent standard deviations from three independent groups. (G) Schematic diagram of gene knockout using negative selection markers. Validation primer locations are also indicated. The corresponding results for F1‐R1, F2‐R2, and F3‐R3 under different conditions are shown in (I). F4‐R4 are insert‐specific primers, and the results are determined based on the presence or absence of amplification, these primers are also used in panel K to identify the unedited genome. (H) Proportions of single‐ and double‐crossover events in transformants (20 colonies per group, 3 groups total) after induction of negative selection markers. Error bars represent standard deviations from three independent groups. (I) Representative PCR gel electrophoresis results using three primer sets corresponding to panel (G). Lane annotations: F1–R1: Lane 1, no single crossover; Lane 2, presence of single crossover; F2–R2: Lane 1, coexistence of wild‐type and single‐crossover; Lane 2, coexistence of wild‐type and double‐crossover; Lane 3, double‐crossover homozygote; F3–R3: Lane 1, absence of wild‐type genome; Lane 2, presence of wild‐type genome. (J) Schematic diagram of replacing the Km^R^ cassette with *pilMNOQ* ^*m*^ in the WTR‐PIL‐KM strain using the T4CROSS method. Validation primers F5–R5 are indicated. (K) Schematic diagram of the same gene replacement using the TRIPLEARM method. The circled numbers indicate the sequence of single‐crossover events, and the purple labels denote the inducers used for selection. (L) Phenotypic or molecular validation results of correctly modified strains under corresponding conditions for both T4CROSS and TRIPLEARM strategies. Growth under *str* ^*R*^ indicates deletion of the *68rpsl* gene cluster or acquisition of mutations conferring resistance. Growth under *cm* ^*R*^ indicates, for the T4CROSS method, retention of the plasmid backbone introduced during the first replacement step, whereas for the TRIPLEARM method it indicates the presence of an unexcised intermediate plasmid backbone. Growth under *ery* ^*R*^ indicates the persistence of a single‐crossover state that has not resolved into a double‐crossover in the T4CROSS method. Representative PCR gel electrophoresis patterns are shown as follows. For T4CROSS: F1–R1: Lane 1, absence of single crossover; Lane 2, presence of single crossover. F2–R2: Lane 1, homozygous final double‐crossover; Lane 2, coexistence of intermediate and final double‐crossover; Lane 3, coexistence of final double‐crossover and single‐crossover. F5–R5: Lane 1, absence of intermediate double‐crossover; Lane 2, presence of intermediate double‐crossover. For TRIPLEARM: F2–R2: Lane 1, homozygous final double‐crossover; Lane 2, coexistence of final double‐crossover and unedited wild‐type state; Lane 3, coexistence of final double‐crossover and the pre‐recombination single‐crossover state corresponding to step K2; Lane 4, coexistence of final double‐crossover and the pre‐recombination single‐crossover state corresponding to step K3. F5–R5: Lane 1, absence of intermediate single‐crossover; Lane 2, presence of intermediate single‐crossover. F4–R4: Lane 1, absence of the original unedited genome; Lane 2, presence of the original unedited genome.

As previously reported (Chen et al. 2021) and confirmed in this study (Figure 3A and Figure S3A), even with the use of two homologous arms, most transformants (*n* = 20, three groups) retained a single crossover state after five passages. To overcome this limitation, we tested a range of potential counter‐selection markers, including: (i) *rpsl*_*12*_, which encodes ribosomal protein S12 (its wild‐type form is toxic in the presence of streptomycin when introduced into a streptomycin‐resistant background) (Takahama et al. 2004); (ii) *sepT*_*2*_, a VapC‐like toxin encoding gene known to be lethal to Syn2973 (Zhou et al. 2019); (iii) *tetA*, which confers tetracycline resistance but increases Ni^2+^ sensitivity (Stavropoulos and Strathdee 2000). The commonly used *sacB* gene in Syn6803, which confers sucrose sensitivity, served as a control (Pelicic et al. 1996). The *tetA* and *sacB* cassettes were inserted into the stable plasmid pSEL under the control of the *P*_*tetA*_ and *P*_*sacB*_ promoters, respectively. *sepT*_*2*_ was placed under a theophylline‐inducible *P*_*trc‐tho*_ switch, resulting in the strains WT‐pSEL‐TetA, WT‐pSEL‐SacB and WT‐pSEL‐SepT_2_ (Table S5). A streptomycin‐resistant strain, WT‐RPSLm, was generated by mutating the native *rpsl*_*12*_ gene (Figure S4). The wild‐type *rpsl*_*12*_ gene from Syn6803 was then introduced into WT‐RPSLm, generating the strain WTR‐pSEL‐68Rpsl. As shown in Figure 3B,C, neither Ni^2+^‐induced expression of *tetA* nor sucrose‐induced *sacB* expression was lethal to Syn2973. In contrast, theophylline‐induced expression of *sepT*_*2*_ significantly inhibited the growth of WT‐pSEL‐SepT_2_ after 48 h (Figure 3D), and streptomycin addition suppressed the growth of WTR‐pSEL‐68Rpsl (Figure 3E). Both systems also successfully eliminated plasmids during two passages post‐induction (Figure 3F, Figure S3B), providing new applications in plasmid cure. We then employed these counter‐selection markers in a two‐step single‐crossover recombination strategy to knock out the *pilMNOQ* gene cluster (Figure 3G). Following a single passage with theophylline induction, ~93.3% of colonies (*n* = 20, three groups) exhibited successful double crossovers, generating a strain WT‐PIL‐KM (Figure 3H,I, with PCR validation shown in Figure S3C).

Next, we aimed to construct marker‐free genetic tools based on the validated counter‐selection markers. As a proof of concept, we replaced the native *pilMNOQ* cluster with a mutant version (*pilMNOQ* ^*m*^) in the WT‐PIL‐KM background (the original *rpsl*_*12*_ gene was first mutated to obtain WTR‐PIL‐KM). The first strategy, named ‘T4CROSS’ (Figure 3J), used two plasmids and four rounds of single‐crossover recombination. In the first step, a cassette containing both a positive selection marker and the *rpsl*_*12*_ counter‐selection marker replaced the kanamycin‐resistance cassette in WTR‐PIL‐KM. Another counter‐selection marker *sepT*_*2*_ was introduced to facilitate the second single crossover at the same time. The resulting strain was chloramphenicol‐resistant and streptomycin‐sensitive. In the second round, a plasmid carrying *pilMNOQ* ^*m*^, an erythromycin resistance marker, and *sepT*_*2*_ was introduced. After selecting transformants on erythromycin, marker‐free replacement of the target gene was achieved via combined induction of streptomycin and theophylline, activating both counter‐selection systems. The second strategy, ‘TRIPLEARM’ (Figure 3K), used a single plasmid and three single‐crossover steps. Here, the cassette containing a positive selection marker and *rpsl*_*12*_ was flanked by two identical homologous arms, enabling excision of the marker in the final step. As with T4CROSS, *sepT*_*2*_ was used to facilitate the intermediate recombination event. As shown in Figure 3L (with PCR validation in Figure S3D,E), both approaches successfully enabled marker‐free genome manipulation. Notably, the resulting strain, WTR‐pilNm, regained natural competence and could be transformed directly using DNA fragments (Figure S3F).

### 3.3 Development of Marker‐Free Gene Knock‐In Tool Based on Modified CRISPR/Cpf1

**FIGURE 4:**
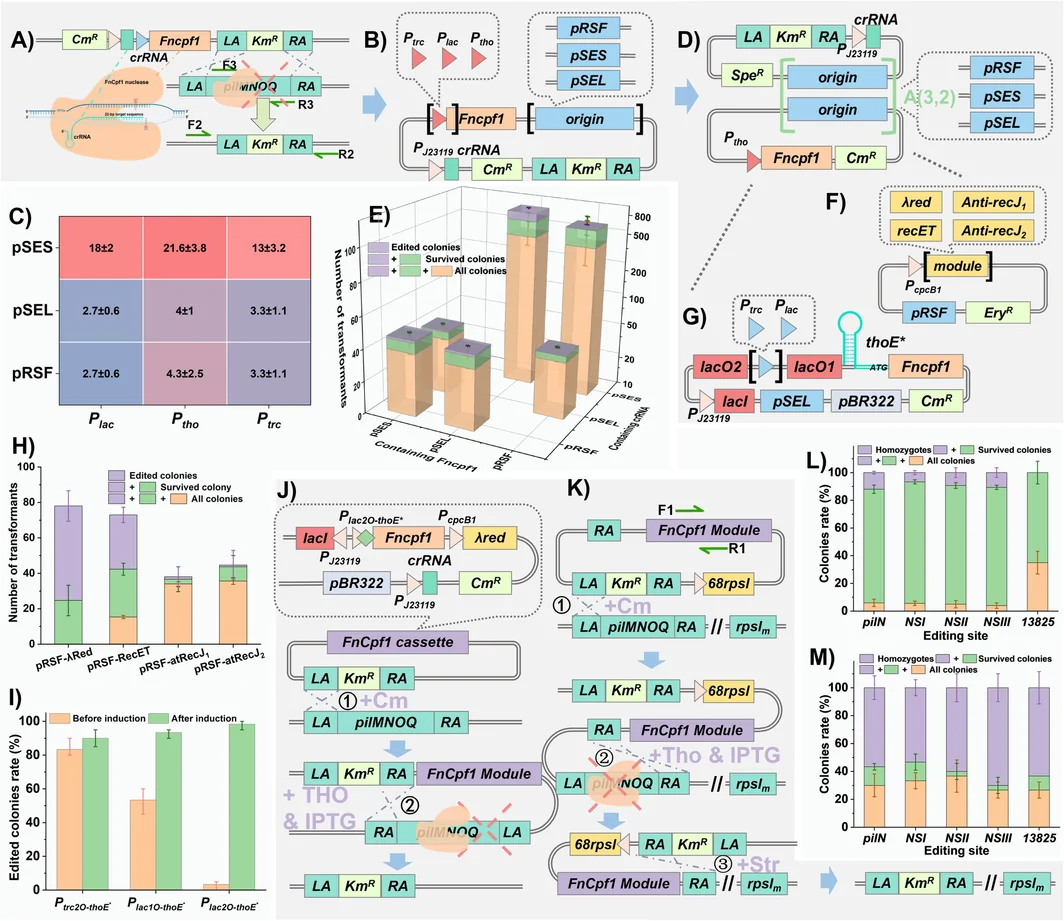
Testing and optimization of the CRISPR/Cpf1 system. Error bars representstandard deviations from three independent experiments. (A) Schematic diagram showing the evaluation of CRISPR/Cpf1 efficiency via knockout of the *pilMNOQ* operon. (B) Diagram of shuttle plasmids carrying the crRNA cassette, homologous insert fragments, and the cpf1 gene driven by different promoters. (C) Transformation efficiency of plasmids described in panel B using an initial culture with OD = 0.5. Numbers indicate the number of colonies formed. (D) Schematic of a two‐plasmid CRISPR system in which the functional modules are separated. The vectors were assembled by selecting and combining two compatible replication origins from a set of three distinct replicons. (E) Number of transformants, survival rate, and editing outcomes after sequential introduction of the FnCpf1 expression plasmid followed by the crRNA plasmid. The transformation efficiency of the first‐step introduction of the plasmid carrying Cpf1 is shown in Figure S3G,F. Schematic of plasmid constructs based on panel D, incorporating either a recombination‐enhancing module or a plasmid designed to repress endogenous recJ expression. (G) Modified system from panel D using a more tightly regulated inducible promoter to control FnCpf1 expression. (H) Number of transformants, survival rate and editing success after introduction of homologous recombination‐enhancing elements in panel F. (20 colonies per group, 3 groups total). (I) Editing efficiency before and after induction of FnCpf1 expression under different inducible systems shown in panel G (20 colonies per group, 3 groups total). (J) Schematic of a one‐plasmid CRISPR strategy in which the CRISPR and recombination modules are placed on a recombination plasmid, which integrates into the target site via a single crossover and is subsequently excised along with the target gene in a second single crossover event. (K) Schematic illustration of the ‘CRISPARM’ strategy. The circled numbers represent the order of single‐crossover events, and the purple annotations indicate the inducers applied during selection. (L) Editing efficiency of the strategy shown in panel J at different genomic loci (20 colonies per group, 3 groups total). *pilN* denotes a pilus structural gene. *NSI*, *NSII* and *NSIII* represent neutral genomic integration sites. *13825* refers to *M744_13825*, which encodes a key enzyme in the glycogen biosynthesis pathway; its deletion markedly impairs cell growth. (M) Editing efficiency of the ‘CRISPARM’ method across various gene loci.

Genome editing using CRISPR/Cpf1 avoids the cytotoxicity associated with Cas9 (Patel et al. 2023) by directly cleaving target sequences, thereby forcing cells to survive through either homology‐directed repair (HDR) or non‐homologous end joining (NHEJ). A CRISPR/Cpf1‐based system has previously been reported in Syn2973 (Ungerer and Pakrasi 2016). Initially, we constructed an all‐in‐one shuttle plasmid based on pRSF‐ori, harbouring *cpf1*, crRNA and repair templates, targeting the *pilMNOQ* cluster (Figure 4A). Three promoters of varying strength—*P*_*lac*_, *P*_*trc*_ and *P*_*tho*_ (specifically P_trc‐thoE*_)—were used to drive *cpf1* expression. However, using our standard transformation conditions (0.5 OD, ~7.25 × 10^7^ cells), no transformants were obtained, suggesting low editing efficiency. Switching the backbone from pRSF‐ori to our modified vectors (pSES, pSEL and pRSF, Figure 4B) did not yield any edited colonies either (Figure 4C). To overcome this limitation, we explored a stepwise transformation strategy by separating *cpf1* and crRNA expression into two different plasmids (Figure 4D). All six possible plasmid combinations using pSES, pSEL and pRSF were evaluated. As shown in Figure 4E, the stepwise strategy significantly improved transformation efficiency, with pSES‐based plasmids generating the highest number of colonies. Notably, over 100 transformants were obtained with combinations of pSES (carrying crRNA) and pSEL or pRSF (carrying *cpf1*). However, most transformants failed to survive subsequent subculturing (Figure 4E), and only a small fraction of the survivors was confirmed to be successfully edited (PCR verification in Figure S5A). We hypothesized that the mismatch between the high DNA cleavage activity of Cpf1 and the low HDR efficiency in Syn2973 was a key bottleneck. Unlike *E. coli* or Syn6803, Syn2973 lacks key recombination‐related genes such as *lexA* and *recD*, which may also contribute to the reduced resistance to DNA damage observed in its close relative Syn7942 (Cassier‐Chauvat et al. 2016).

To balance DNA cleavage with recombination, we utilized a two‐pronged approach: (i) Enhancing recombination via introduction of additional systems such as λ‐red (Yu et al. 2000) and RecET (Wang et al. 2016), and inhibition of endogenous *recJ*, which was previously shown to increase HDR efficiency in Syn2973 (Racharaks et al. 2021) (Figure 4F); and (ii) Implementing tighter control of *cpf1* expression using a tandem inducible system responsive to IPTG and theophylline (Figure 4G). As shown in Figure 4H, both λ‐Red and RecET improved editing efficiency, with λ‐Red being more effective (PCR results in Figure S5B). Moreover, combining λ‐Red with *cpf1* under the dual‐inducible promoter *P*_lac2O‐thoE*_ allowed precise control: no editing occurred without induction, whereas clear editing was observed following induction with 2 mM theophylline and 1 mM IPTG (Figure 4I, Figure S6A). However, even with this strategy, homozygous edited colonies were not obtained (Figure S5C), likely due to instability of the pSES‐based plasmid or insufficient recombination efficiency. To further improve outcomes, we employed an integrative plasmid approach (Figure 4J), wherein the CRISPR/Cpf1 cassette was integrated into the genome via an initial single crossover. Induction of CRISPR/Cpf1 then promoted a second crossover, achieving marker‐free editing by excising the target gene and editing cassette itself together. This approach was validated by targeting *pilMNOQ*, three neutral sites (NSI, NSII, NSIII), and gene *M744_13 825* (*glgC* involving glycogen biosynthesis), which affects growth upon deletion (Wang et al. 2023) (Figure 4L, Figure S6B). Homozygous transformants were obtained for all non‐essential targets (~10% efficiency; Figure S6D). However, for *M744_13 825*, no homozygotes were recovered, potentially due to the loss of Cpf1 function before complete homozygosity was achieved. To further optimize this approach, we combined the enhanced CRISPR/Cpf1 tool with our previously established ‘TRIPLEARM’ method, creating a hybrid strategy named ‘CRISPARM’ Here (Figure 4K), both the Cpf1 cassette and the Rpsl_12_ counter‐selection marker were flanked by identical homologous arms. Dual induction with IPTG and theophylline, combined with positive selection for resistance, enabled the isolation of edited strains. The Cpf1 cassette was subsequently removed through streptomycin‐based counter‐selection. Using an 8 kb GOI to evaluate the system, we achieved homozygous editing in all target loci with > 50% efficiency (Figure 4M and Figure S6C,E). Approximately 30% of transformants exhibited unedited outcomes, mainly due to survival failure during the sub‐induction process. These were likely caused by unintended single crossover events due to the presence of three homologous arms, which is discussed further in the subsequent sections.

### 3.4 Standardization and Comparison of Scarless Operation Methods

**FIGURE 5:**
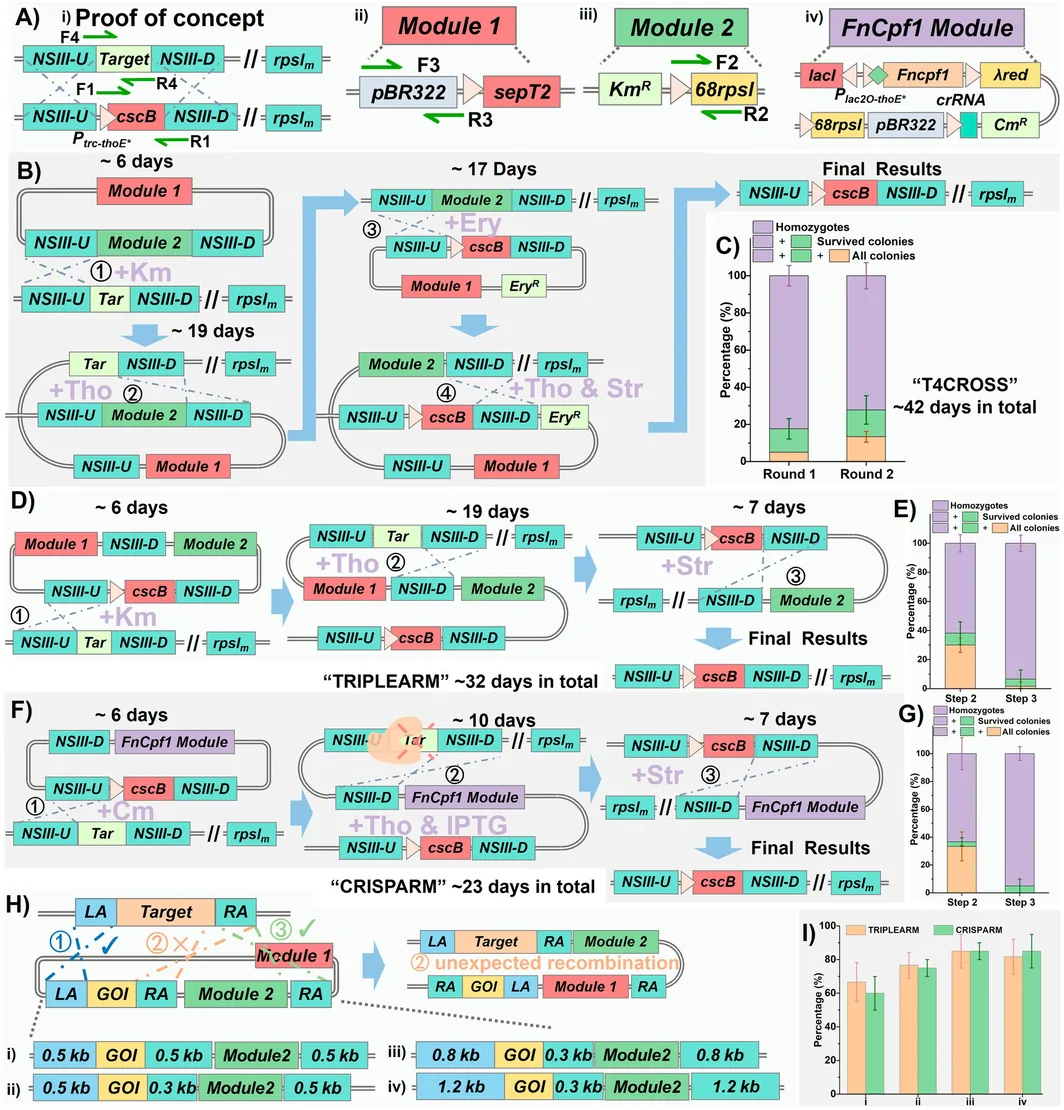
Integration of the *cscB* expression cassette into Syn2973 using three marker‐free genome editing strategies for sucrose secretion. Error bars represent standard deviations from three independent groups. (A) Schematic overview of introducing *cscB* into Syn2973 to construct the WTR‐B3 strain, along with simplified diagram legends used in other subpanels. The positions of validation primers are also indicated in the schematic. (B) Construction of WTR‐B3 using the T4CROSS method. The circled numbers indicate the sequence of single‐crossover events, the adjacent purple labels denote the additives used for induction and selection, and the time indicates the approximate duration required for each step. (C) Proportions of survived colonies and homozygotes obtained in the first and second rounds of transformation using the T4CROSS strategy. (D) Construction of WTR‐B3 using the TRIPLEARM method. (E) Proportions of survived colonies and homozygotes obtained in the first and second rounds of transformation method TRIPLEARM (20 colonies per group, 3 groups total). (F) Construction of WTR‐B3 using the CRISPARM method. (G) Survival and homozygosity rates in the second and third recombination steps using CRISPARM (20 colonies per group, 3 groups total). (H) Schematic representation of the possible homologous recombination pathways during the first step of the TRIPLEARM and CRISPARM strategies, showing two correct recombination modes and one incorrect mode. The incorrect recombination pathway is illustrated in detail on the right. The lower panel presents an optimized strategy to improve recombination specificity by adjusting the lengths of the three homologous arms. In this design, homologous arms that enable correct recombination are grouped with identical lengths, whereas those leading to incorrect recombination are assigned to a separate group. (I) Effect of different homologous arm length combinations shown in panel (H) on the survival rate of transformants after induction.

To facilitate broader application of the genome editing tools developed above, we standardized the entire manipulation workflow and compared the editing efficiency of each method. As a model case, we introduced the *cscB* cassette encoding sucrose permease into the WT‐RPSLm strain to enable sucrose secretion (Figure 5A).

For the first method, ‘T4CROSS’ (Figure 5B), a plasmid designed to replace the NSIII site with both a positive and a counter‐selection cassette was first introduced. Following approximately 6 days of selection, individual colonies were transferred to shake flasks for expansion. Inducers and antibiotics were then added to trigger the second single‐crossover recombination event. After approximately three passages, homozygous mutants were verified by qRT‐PCR. This step took around 19 days in total, with 83.3% of the colonies identified as homozygous (Figure 5C). Subsequently, a second plasmid carrying the *cscB* cassette was introduced into the verified homozygotes. After two further rounds of single‐crossover recombination, induced by streptomycin and theophylline, the final sucrose‐producing strain WTR‐B3—free of any selection markers—was obtained. This second editing phase required approximately 17 days, yielding a final positive colony rate of 73.3% (*n* = 20, three groups) (Figure 5C). Overall, the T4CROSS method required about 42 days to complete the full editing cycle.

For the ‘TRIPLEARM’ (Figure 5D) and ‘CRISPARM’ (Figure 5F) methods, a single plasmid targeting the NSIII site was constructed for each approach. Selection for the first integration event was performed using kanamycin or chloramphenicol, respectively, and both methods required approximately 6 days for this step. In the TRIPLEARM workflow, the second single‐crossover recombination was induced using theophylline after colonies growing to mid‐log phase in shaking flasks, followed by three serial passages to enrich for homozygous mutants, which were again confirmed by qRT‐PCR. In contrast, for the CRISPARM method, IPTG and theophylline were added directly after transferring the selected colonies into flasks, enabling faster induction. The TRIPLEARM approach required approximately 19 days, with a final homozygous positive rate of 65.0% (Figure 5E). The CRISPARM method took only 10 days, yielding a positive rate of 62.7% (Figure 5G). In both approaches, streptomycin was used in the final step to trigger a third single‐crossover recombination event, leading to the removal of editing cassettes and recovery of marker‐free strains. The entire workflows required approximately 32 days for TRIPLEARM and 23 days for CRISPARM. It is worth noting that both TRIPLEARM and CRISPARM require the simultaneous presence of three homologous arms, which introduces the potential for unintended recombination events due to three possible crossover sites (Figure 5H). To optimize recombination directionality and efficiency, we systematically adjusted the lengths of the three homologous arms. The best performance was observed when the two terminal arms were extended to 800 bp and the central arm was shortened to 300 bp. Under these conditions, the editing efficiency reached approximately 85.0% (Figure 5I), demonstrating that fine‐tuning arm lengths can effectively improve recombination outcomes.

To rigorously evaluate the genetic stability of the engineered strains, we compared the retention of heterologous genes (*lacZ* and *gusA* encoding beta‐D‐glucuronidase) in strains constructed by either traditional single‐crossover or the CRISPARM‐mediated marker‐less double‐crossover method. Ten independent transformants from each group were subjected to continuous passaging in BG11 medium without antibiotic selection for 35 days. The enzymatic activities of LacZ and GusA were quantitatively measured at 7‐day intervals. The results showed a clear divergence in functional stability (Figure S7). In single‐crossover‐derived strains, the enzymatic activities of LacZ and GusA began to decrease significantly after the first week, eventually dropping to 23.80% and 20.94% of their initial levels by day 35, respectively. Conversely, the CRISPARM‐derived marker‐less strains exhibited remarkable stability, with both LacZ and GusA activities remaining constant over 35 passages, demonstrating the genetic robustness necessary for long‐term biotechnological applications via CRISPARM.

To rigorously characterize the performance of the three strategies and address potential selection biases, we compared the efficiency of T4CROSS and TRIPLEARM to that of CRISPARM across multiple genomic loci (NSI, NSIII, *pilN* and *M744_13 825*) (Figure S9). We observed that for standard neutral sites such as NSI, although CRISPARM resulted in a lower survival rate compared to T4CROSS, the final yield of homozygous clones relative to the starting population was comparable across all methods, with CRISPARM being the most time‐efficient. Crucially, for difficult loci like M744_13 825 whose deletion would block glycogen biosynthesis and inhibit growth, T4CROSS and TRIPLEARM methods failed to achieve full chromosome segregation within a practical experimental timeframe. For these loci, the high selection pressure exerted by CRISPARM was essential to eliminate wild‐type alleles and drive the population toward homozygosity. These data provide a comprehensive map of the strengths and boundaries of each tool, demonstrating that CRISPARM's efficiency is a result of robust selection rather than a statistical artefact.

To address whether the developed T4CROSS, TRIPLEARM and CRISPARM strategies could be generalized to other microbes beyond Syn2973, we extended our evaluation to three additional species: the model polyploid cyanobacterium *S. elongatus* PCC 7942, the model bacterium *E. coli* , and the industrial bacterium *G. oxydans* . Specifically, we targeted the NSI neutral site, the *lacZ* gene, and the GOX0013 gene for replacement with a 1‐kb exogenous DNA fragment, respectively in three strains. Our results demonstrated that all three strategies were successfully implemented in these diverse hosts, consistently yielding homozygous or positive transformants (Figure S9). Notably, the CRISPARM method consistently outperformed T4CROSS and TRIPLEARM in terms of positive colony frequency across all tested species, reinforcing its role as a high‐efficiency tool for rapid genome editing. These findings collectively demonstrate that our iterative genome engineering platform is not strain‐specific but rather a versatile toolkit applicable to a broad range of prokaryotic organisms with varying genomic characteristics.

### 3.5 Iterative Marker‐Free Genetic Manipulations Enabled High Production of Sucrose in Syn2973

**FIGURE 6:**
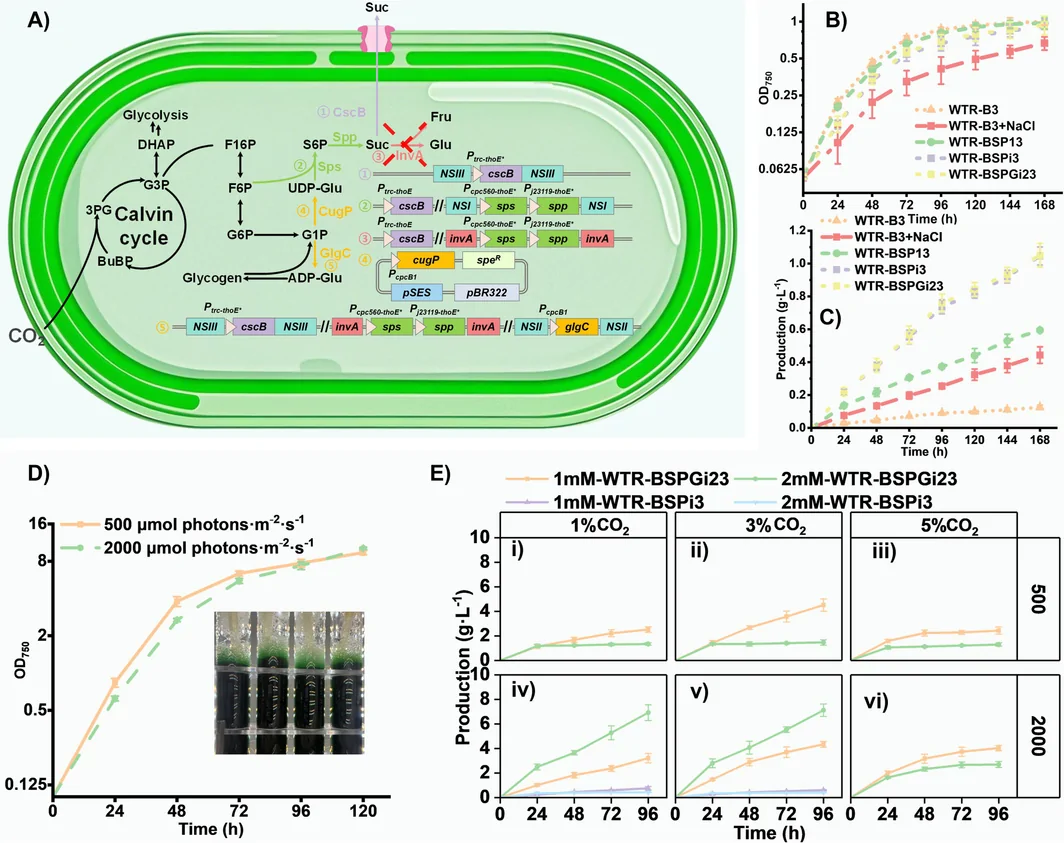
Sucrose production through iterative metabolic engineering of Syn2973. Error bars represent standard deviations from three independent replicates. (A) Schematic diagram of the sucrose synthesis and degradation pathways in Syn2973, along with the sequential engineering steps applied in this study. (B) Growth curves of sucrose‐producing strains WTR‐B3, WTR‐BSP13, WTR‐BSPi3, WTR‐BSPGi23 and WTR‐B3 cultured in the presence of 150 mM NaCl. (C) Sucrose production of the strains listed in panel B under 150 mM NaCl stress. (D) Growth curve of WTR‐BSPGi23 cultivated in a bubble column photobioreactor with 3% CO_2_ under different light intensities. (E) Sucrose yields of WTR‐BSPi3 and WTR‐BSPGi23 under varying conditions, including 1%, 3%, or 5% CO_2_ concentrations, theophylline induction at 1 mM or 2 mM, and light intensities of 500 or 2000 μmol photons m^−2^ s^−1^.

As described above, a sucrose‐secreting strain, WTR‐B3, was successfully constructed. When 150 mM NaCl was added to the medium, sucrose production was significantly enhanced in Syn2973, reaching 662.2 mg L^−1^ OD^−1^, compared to only 125.3 mg L^−1^ OD^−1^ under salt‐free conditions (Figure 6B,C). However, this increase in production came at the cost of inhibited cell growth. To improve sucrose production without salt stress, we applied the developed ‘CRISPARM’ method to perform iterative marker‐free genetic modifications. The key rate‐limiting steps in the cyanobacterial sucrose synthesis pathway are catalyzed by sucrose‐phosphate synthase (Sps) and sucrose‐phosphate phosphatase (Spp). These enzymes are strongly upregulated during salt induction and are considered primary targets for enhancing sucrose biosynthesis. To bypass the requirement for NaCl, the *sps* and *spp* genes from Syn6803 were overexpressed in WTR‐B3 by integrating their expression cassettes into the NSI site, generating strain WTR‐BSP13. As a result, sucrose production increased significantly even in the absence of salt, while cell growth was also restored (Figure 6B,C). The sucrose yield reached 610.5 mg L^−1^ OD^−1^, comparable to that of WTR‐B3 under NaCl induction, suggesting that overexpression of *sps* and *spp* can mimic the effect of salt stress on sucrose production. Next, to further enhance sucrose accumulation, we targeted *invA*, a gene encoding invertase responsible for hydrolyzing sucrose into glucose and fructose. The *sps* and *spp* cassette was integrated directly into the *invA* locus, generating WTR‐BSPi3. This strain exhibited an additional ~40% increase in sucrose production, albeit with a slight reduction in growth (Figure 6B,C).

To further boost sucrose synthesis, we explored increasing the supply of its precursors—fructose‐6‐phosphate (F6P) and UDP‐glucose (UDP‐Glc). Given F6P's central role in carbon fixation and glycolysis, it was unlikely to be a limiting substrate. Therefore, we focused on enhancing UDP‐Glc synthesis by overexpressing *cugP*, a non‐GalU‐type UDP‐glucose pyrophosphorylase from Syn6803, in WTR‐BSPi3. We attempted chromosomal integration of *cugP* at the NSII site, but no positive transformants were obtained across three independent trials. Alternatively, we used the high‐efficiency free‐shuttle plasmid pSES to introduce the *cugP* cassette. Although transformants were initially obtained, they failed to survive after induction in shake flask cultivation. Interestingly, despite its apparent contradiction, previous studies have reported that overexpression of *glgC*—encoding ADP‐glucose pyrophosphorylase, a key enzyme in glycogen biosynthesis—can enhance sucrose production (Qiao et al. 2018). It is hypothesized that increased glycogen synthesis diverts more carbon flux from glycolysis toward glucose‐1‐phosphate (G1P), thereby promoting UDP‐Glc generation. We thus overexpressed *glgC* from Syn6803 in WTR‐BSPi3 by targeting the NSII site, resulting in the strain WTR‐BSPGi23. Under standard shake flask conditions, *glgC* overexpression did not significantly affect either growth or sucrose yield (Figure 6B,C).

To further evaluate performance under more physiologically relevant conditions, we conducted bubble‐column photobioreactor cultivation under various combinations of environmental factors. Specifically, we tested both WTR‐BSPi3 and WTR‐BSPGi23 under three CO_2_ concentrations (1%, 3% and 5%), two light intensities (500 and 2000 μmol photons m^−2^ s^−1^), and two concentrations of theophylline (1 and 2 mM) as inducers. The results showed that WTR‐BSPi3 produced detectable levels of sucrose only under 1% and 3% CO_2_ at high light intensity (2000 μmol photons m^−2^ s^−1^), but its yield was lower than that observed in shake flasks (Figure 6E). In contrast, WTR‐BSPGi23, overexpressing *glgC*, exhibited a significant advantage under aerated conditions. Under optimized conditions—3% CO_2_, 2000 μmol photons m^−2^ s^−1^ light, and 2 mM theophylline—this strain reached a sucrose titre of 7.12 g L^−1^ after 4 days, corresponding to an average productivity of 1.79 g L^−1^ day^−1^.

## 4 Discussion

Single‐crossover events during homologous recombination observed in microbes pose risks to strain stability and hinder iterative genome engineering. In our previous work, we established a suite of regulatory elements tailored for Syn2973, including constitutive and inducible promoters, as well as gene regulation systems (Li et al. 2018). Gene editing and gene silencing tools have also been developed in recent years (Ungerer and Pakrasi 2016; Wendt et al. 2016). However, we find these tools are still insufficient for high‐efficiency, iterative genome engineering due to three key limitations: (i) the absence of stable high‐copy‐number shuttle plasmids, which necessitates chromosomal integration of exogenous genes in most cases; (ii) a strong bias toward single crossover events over double crossovers, even when two homologous arms are provided, complicating the identification of correctly modified strains; and (iii) the lack of efficient marker‐free editing strategies, limiting the number of sequential genetic modifications that can be performed.

Among currently available tools, RSF1010 is the only known broad‐host‐range replicon capable of stable maintenance in several cyanobacteria, especially Syn6803 and *Nostoc* sp. PCC 7120 (Mermet‐Bouvier et al. 1993; Bharadwaj et al. 2023). Although previous studies have employed RSF1010 for genetic manipulation in Syn2973, our findings reveal its transformation efficiency in this strain is low, requiring extensive screening to identify positive clones. We speculate that this inefficiency stems from its low plasmid copy number. Deletion of the *repF* gene partially alleviated this issue but did not fully resolve the instability, suggesting that RSF1010's replication module does not function optimally in Syn2973. To address this, we developed two novel free‐replicating plasmids, pSES and pSEL, by fusing endogenous replication elements from Syn2973 with *E. coli* replicons. These plasmids exhibited satisfactory transformation efficiency and stable maintenance. However, the presence of homologous endogenous plasmids may compete with the introduced plasmids, reducing their stability. In future work, eliminating these endogenous plasmids—if non‐essential—could enhance plasmid maintenance, particularly for pSES. Furthermore, although pSEL exhibited reliable replication, its large replication module complicates plasmid construction, suggesting future efforts should focus on streamlining the replication region.

Another major challenge is Syn2973's preference for single crossover recombination, which compromises the genetic stability of engineered strains and necessitates laborious screening to identify double crossover events. Although modifying the host genome to overcome this bias remains difficult, we developed two counter‐selection marker systems that enable a two‐step single crossover strategy to achieve functionally equivalent double crossovers. Despite increasing construction time, this approach is far more efficient than blind screening. Previous studies by Chen et al. introduced counter‐selection systems including *SYNPCC7002_G0085* from *Synechococcus* sp. PCC 7002 and *mazF* from *E. coli* (Chen et al. 2021). Our work complements these systems by expanding the available toolkit. Through comparative analysis of recombination systems across cyanobacteria, we also identified the absence of key recombinases in Syn2973 as a possible contributor to its recombination inefficiency. While introducing exogenous recombinases moderately improved recombination outcomes, the improvement was limited. In future studies, a systems biology approach could be employed to dissect the genetic basis of recombination bias in Syn2973, allowing for rational complementation or rewiring of its recombination machinery.

To date, very few studies have reported sequential, marker‐free genome editing in Syn2973. To our knowledge, only Ungerer et al. have used CRISPR/Cpf1 to simultaneously mutate three genes (Ungerer and Pakrasi 2016). Building on our newly developed free‐replicating plasmids and negative selection systems, we established three distinct strategies for marker‐free genetic manipulation: (i) T4CROSS, which uses two plasmids and two transformation steps, enabling both point mutations and gene insertions with high precision. (ii) TRIPLEARM, a single‐step transformation approach ideal for gene insertions. However, it is not suitable for point mutations or fine‐tuned sequence changes. Additionally, the use of three homologous arms increases the risk of incorrect recombination, which can be mitigated by optimizing homologous arm lengths. (iii) CRISPRARM, the most efficient and rapid approach, but with some limitations. Its large plasmid size presents cloning challenges, and as it is an integrative system, the post‐editing removal of the editing plasmid is more complex than that of free‐replicating systems. Future directions include the use of smaller Cas proteins, such as IscB (Xiao et al. 2025), and further optimization of free‐replicating plasmids to combine efficiency with ease of use. Collectively, the systems developed in this study not only address several key challenges in Syn2973 engineering but also provide a scalable foundation for future multi‐gene and pathway‐level genome engineering in this promising photosynthetic chassis.

In this study, a marker‐free sucrose‐producing strain was constructed using the methods described above. Following fermentation optimization, a final sucrose titre of 7.12 g L^−1^ was achieved within 4 days, approaching the current highest reported levels of 8 g L^−1^ (4 days, salt‐induced) (Lin et al. 2020) and 8.7 g L^−1^ (21 days) (Song et al. 2016). These results demonstrate the effectiveness of the proposed strategies for constructing Syn2973‐based cell factories. In the future, further investigation into the interplay between sucrose and glycogen biosynthesis could enable more precise carbon flux redistribution toward sucrose production, while culture condition optimization and increased cell density could further enhance sucrose yields.

## Funding

This research was supported by grants from the National Key Research and Development Program of China (Grant no. 2024YFA0919700), the National Natural Science Foundation of China (Grant nos. 32371486 and 32270091), the Natural Science Foundation of Tianjin (Grant no. 23JCYBJC01680), and the Haihe Laboratory of Sustainable Chemical Transformations.

## Conflicts of Interest

The authors declare no conflicts of interest.

## Supporting information


**Data S1:** Supporting Information.

**Figure S1:** Standard calibration curve showing the relationship between sucrose concentration and peak area as measured by HPLC with refractive index detection (RID).
**Figure S2:** Representative plate images showing transformant growth for NSI, pRSF‐ori, pRSF, pSES‐ori, pSES, pSEL‐ori and pSEL at initial DNA input amounts of 0.01 and 0.1.
**Figure S3:** (A) PCR verification of single colonies isolated from each generation shown in Figure 3A using primers F2 and R2 from Figure 3. The leftmost lane of the gel represents the DNA ladder (marker), with bands migrating from top to bottom at 20 kb, 10 kb, 7 kb, 5 kb, 4 kb, 3 kb, 2 kb, 1.5 kb, 1 kb, 700 bp, 500 bp, 400 bp, 300 bp, 200 bp and 75 bp, respectively. The bolded values indicate the brighter reference bands. The position and size distribution of the marker remain identical across all subsequent gel images. (B) Growth of single colonies on BG11 plates supplemented with kanamycin after plasmid curing via sepT_2_ and rpsl selection. (C) PCR verification of selected single colonies from Figure 3H using primers F1‐R1, F2‐R2 and F3‐R3 from Figure 3. (D) PCR verification of WTR‐pilNm strains obtained using the ‘T4CROSS’ strategy in Figure 3L, with primers F2R2, F1R1 and F5R5 from Figure 3. (E) PCR verification of WTR‐pilNm strains obtained using the ‘TRIPLEARM’ strategy in Figure 3L, with primers F2‐R2, F4‐R4 and F5‐R5 from Figure 3. (F) Natural transformation results of WTR‐pilNm strains obtained by the ‘T4CROSS’ and ‘TRIPLEARM’ strategies. (G) Number of transformants in the first step of FnCpf1 introduction using plasmids pSES, pSEL and pRSF, as shown in Figure 4E.
**Figure S4:** (A) Introduction of the mutation via homologous double crossover. (B) Unexpected mutations observed during the process depicted in panel A. (C) Growth of the resulting strains from panel A on solid medium supplemented with streptomycin. (D) Transformation efficiency and homozygosity of rpsl12 point mutations using the strategies described in panels A, E and H. (E) Schematic diagram of point mutation introduction via a two‐step strategy: homologous single crossover with a positive selection marker, followed by homologous double crossover using rpsl12 as a negative selection marker. (F) Growth of strains obtained using the method in panel E on streptomycin‐containing medium. (G) Growth of strains obtained using the method in panel E on chloramphenicol‐containing medium. (H) Modified version of the strategy in panel E, incorporating SepT2 as a negative selection marker to facilitate the second crossover. (I) Growth of strains obtained using the method in panel H on streptomycin‐containing medium. (J) Growth of strains obtained using the method in panel H on chloramphenicol‐containing medium.
**Figure S5:** (A) Verification of survived transformants from Figure 4E. (B) Verification of survived transformants from Figure 4H. (C) Verification of surviving transformants from Figure 4I before and after induction; lanes to the left of the marker indicate samples before induction, and lanes to the right indicate samples after induction.
**Figure S6:** (A) Number of transformants obtained in Figure 4I. (B) Number of transformants obtained in Figure 4L. (C) Number of transformants obtained in Figure 4M. (D) PCR verification of surviving transformants from Figure 4L using gene‐specific F2‐R2 primers as indicated in Figure 4. (E) PCR verification of surviving transformants from Figure 4M using gene‐specific F2‐R2 primers as indicated in Figure 4.
**Figure S7:** Quantitative assessment of long‐term genetic stability in Syn2973. Comparison of functional stability between strains constructed via traditional single‐crossover (SCO) and CRISPARM‐mediated marker‐less double‐crossover (DCO). (A) Enzymatic activities of LacZ and GusA in SCO strains over 35 days of non‐selective passaging. (B) Enzymatic activities of LacZ and GusA in DCO‐derived marker‐less strains under identical conditions. Strains were passaged daily, and activities were measured every 7 days. LacZ activity is expressed in Miller units, and GusA activity is expressed as specific activity (nmol pNP/min/mg protein). Data represent the mean ± SD of 10 independent biological replicates (*n* = 10).
**Figure S8:** Validation of T4CROSS and TRIPLEARM strategies for large‐fragment replacement at various genomic loci. A series of 8‐kb arbitrary DNA fragments were used to replace five distinct genomic loci—NS I, NS II, NS III, pilN and M744_13 825—to evaluate the efficiency of the developed strategies. (A–E) Targeted replacement of the five respective loci using the T4CROSS strategy. (F–J) Targeted replacement of the five respective loci using the TRIPLEARM strategy. Successful integration and homozygous segregation were verified by colony PCR and/or sequencing.
**Figure S9:** Validation of platform generality across diverse microbial hosts. The performance of T4CROSS, TRIPLEARM and CRISPARM strategies was evaluated in *Synechococcus elongatus* PCC 7942 (A–C), *Escherichia coli* (D–F), and *Gluconobacter oxydans* (G–I) by replacing target loci with a 1‐kb exogenous DNA fragment. (A–C) Targeted engineering of the NS I neutral site in the polyploid *S. elongatus* PCC 7942. (D–F) Targeted deletion of the lacZ gene in *E. coli* . (G–I) Targeted disruption of the GOX0013 gene in *G. oxydans* . For each host, the editing efficiency was determined by analysing 60 randomly selected colonies (‘All colonies’). ‘Homozygotes/Positive’ refers to homozygous double‐crossover transformants in the polyploid *S. elongatus* PCC 7942 or confirmed positive transformants in the monoploid *E. coli* and *G. oxydans* . ‘Survived colonies’ denotes the fraction of transformants that remained viable throughout the iterative passaging and induction process. Data are presented as the mean ± SD from three independent biological replicates.

**Table S1:** The plasmids used in this article, the methods employed for their construction and the corresponding primers are presented. The templates marked in red are the existing ones in the laboratory, while those marked in green are the synthesized ones.
**Table S2:** Sequences of key genes.
**Table S3:**. Primer sequences of qRT‐PCR.
**Table S4:**. Backbone sequences of plasmids.
**Table S5:**. Descriptions of strains used or constructed in this study.

## Data Availability

The authors declare that all data supporting the findings of this study are available within the paper and its Supporting Information files. All customized plasmids developed in this work, including backbones and intermediate constructs, are described in detail in the Supporting Information Files, which contain over 159 annotated SnapGene‐compatible maps (.dna format). Sequences for the core set of 40 functional plasmids have been deposited in the NCBI GenBank database under the accession numbers [PZ357609] to [PZ357648]. All other biological materials and data are available from the corresponding authors upon reasonable request.
