# Supplementary material for: Iterative Genome Engineering Platform Enables Efficient Sucrose Biosynthesis From CO2 in Photosynthetic Synechococcus elongatus UTEX 2973
Source: Plant Biotechnol J. 2026 Jun 15;24(10):5284–99. doi: 10.1111/pbi.70702 (PMC13398943; doi:10.1111/pbi.70702)
Supplement: Supplementary file 1 [file PBI-24-5284-s002.docx]

**Fig. S1.** Standard calibration curve showing the relationship between sucrose concentration and peak area as measured by HPLC with refractive index detection (RID).

**Fig. S2.** Representative plate images showing transformant growth for NSI, pRSF-ori, pRSF, pSES-ori, pSES, pSEL-ori, and pSEL at initial DNA input amounts of 0.01 and 0.1.

**Fig. S3. A)** PCR verification of single colonies isolated from each generation shown in Fig. 3A using primers F2 and R2 from **Fig. 3**. The leftmost lane of the gel represents the DNA ladder (marker), with bands migrating from top to bottom at 20 kb, 10 kb, 7 kb, **5 kb**, 4 kb, 3 kb, 2 kb, **1.5 kb**, 1 kb, 700 bp, **500 bp**, 400 bp, 300 bp, 200 bp, and 75 bp, respectively. The bolded values indicate the brighter reference bands. The position and size distribution of the marker remain identical across all subsequent gel images. **B)** Growth of single colonies on BG11 plates supplemented with kanamycin after plasmid curing via *sepT_2_* and *rpsl* selection. **C)** PCR verification of selected single colonies from Fig. 3H using primers F1-R1, F2-R2, and F3-R3 from Fig. 3. **D)** PCR verification of WTR-pilNm strains obtained using the "T4CROSS" strategy in Fig. 3L, with primers F2R2, F1R1, and F5R5 from Fig. 3. **E)** PCR verification of WTR-pilNm strains obtained using the "TRIPLEARM" strategy in Fig. 3L, with primers F2-R2, F4-R4, and F5-R5 from Fig. 3. **F)** Natural transformation results of WTR-pilNm strains obtained by the "T4CROSS" and "TRIPLEARM" strategies. **G)** Number of transformants in the first step of FnCpf1 introduction using plasmids pSES, pSEL, and pRSF, as shown in Fig. 4E.

**Fig. S4. A)** Introduction of the mutation via homologous double crossover. **B)** Unexpected mutations observed during the process depicted in panel A. **C)** Growth of the resulting strains from panel A on solid medium supplemented with streptomycin. **D)** Transformation efficiency and homozygosity of rpsl12 point mutations using the strategies described in panels A, E, and H. **E)** Schematic diagram of point mutation introduction via a two-step strategy: homologous single crossover with a positive selection marker, followed by homologous double crossover using rpsl12 as a negative selection marker. **F)** Growth of strains obtained using the method in panel E on streptomycin-containing medium. **G)** Growth of strains obtained using the method in panel E on chloramphenicol-containing medium. **H)** Modified version of the strategy in panel E, incorporating SepT2 as a negative selection marker to facilitate the second crossover. **I)** Growth of strains obtained using the method in panel H on streptomycin-containing medium. J) Growth of strains obtained using the method in panel H on chloramphenicol-containing medium.

**Fig. S5 A)** Verification of survived transformants from Fig. 4E. **B)** Verification of survived transformants from Fig. 4H. **C)** Verification of surviving transformants from Fig. 4I before and after induction; lanes to the left of the marker indicate samples before induction, and lanes to the right indicate samples after induction.

**Fig. S6. A)** Number of transformants obtained in Fig. 4I. **B)** Number of transformants obtained in Fig. 4L. **C)** Number of transformants obtained in Fig. 4M. **D)** PCR verification of surviving transformants from Fig. 4L using gene-specific F2-R2 primers as indicated in Fig. 4. **E)** PCR verification of surviving transformants from Fig. 4M using gene-specific F2-R2 primers as indicated in Fig. 4.

**Fig. S7**. Quantitative assessment of long-term genetic stability in Syn2973. Comparison of functional stability between strains constructed via traditional single-crossover (SCO) and CRISPARM-mediated marker-less double-crossover (DCO). (A) Enzymatic activities of LacZ and GusA in SCO strains over 35 days of non-selective passaging. (B) Enzymatic activities of LacZ and GusA in DCO-derived marker-less strains under identical conditions. Strains were passaged daily, and activities were measured every 7 days. LacZ activity is expressed in Miller units, and GusA activity is expressed as specific activity (nmol pNP/min/mg protein). Data represent the mean ± SD of 10 independent biological replicates (n=10).

**Fig. S8. Validation of T4CROSS and TRIPLEARM strategies for large-fragment replacement at various genomic loci.** A series of 8-kb arbitrary DNA fragments were used to replace five distinct genomic loci—NS I, NS II, NS III, *pilN*, and *M744_13825*—to evaluate the efficiency of the developed strategies. (A–E) Targeted replacement of the five respective loci using the T4CROSS strategy. (F–J) Targeted replacement of the five respective loci using the TRIPLEARM strategy. Successful integration and homozygous segregation were verified by colony PCR and/or sequencing.

**Fig. S9. Validation of platform generality across diverse microbial hosts.** The performance of T4CROSS, TRIPLEARM, and CRISPARM strategies was evaluated in *Synechococcus elongatus* PCC 7942 (A–C), *Escherichia coli* (D–F), and *Gluconobacter oxydans* (G–I) by replacing target loci with a 1-kb exogenous DNA fragment. (A–C) Targeted engineering of the NS I neutral site in the polyploid *S. elongatus* PCC 7942. (D–F) Targeted deletion of the lacZ gene in *E. coli*. (G–I) Targeted disruption of the GOX0013 gene in *G. oxydans*. For each host, the editing efficiency was determined by analyzing 60 randomly selected colonies ("All colonies"). "Homozygotes/Positive" refers to homozygous double-crossover transformants in the polyploid *S. elongatus* PCC 7942 or confirmed positive transformants in the monoploid *E. coli* and *G. oxydans*. "Survived colonies" denotes the fraction of transformants that remained viable throughout the iterative passaging and induction process. Data are presented as the mean ± SD from three independent biological replicates.

**Table S5**. Descriptions of strains used or constructed in this study.
